# Interobserver variability of image-derived arterial blood SUV in whole-body FDG PET

**DOI:** 10.1186/s13550-019-0486-9

**Published:** 2019-03-04

**Authors:** Frank Hofheinz, Jens Maus, Sebastian Zschaeck, Julian Rogasch, Georg Schramm, Liane Oehme, Ivayla Apostolova, Jörg Kotzerke, Jörg van den Hoff

**Affiliations:** 1 PET Center, Institute of Radiopharmaceutical Cancer Research, Helmholtz-Zentrum Dresden-Rossendorf, Bautzner Landstraße 400, Dresden, Germany; 2 Charité – Universitätsmedizin Berlin, corporate member of Freie Universität Berlin, Humboldt-Universität zu Berlin, and Berlin Institute of Health, Department of Radiation Oncology, Berlin, Germany; 3 Berlin Institute of Health (BIH), Anna-Louisa-Karsch 2, Berlin, 10178 Germany; 4 Charité – Universitätsmedizin Berlin, corporate member of Freie Universität Berlin, Humboldt-Universität zu Berlin, and Berlin Institute of Health, Department of Nuclear Medicine, Berlin, Germany; 5 Division of Nuclear Medicine, Department of Imaging and Pathology, KU/UZ Leuven, Leuven, Belgium; 6 Klinik und Poliklinik für Nuklearmedizin, Universitätsklinikum Carl Gustav Carus, Dresden, Germany; 7 Zentrum für Radiologie und Endoskopie, Abteilung für Nuklearmedizin, Universitätsklinikum Hamburg-Eppendorf, Hamburg, Germany

**Keywords:** PET, FDG, Quantification, SUV, SUR, Blood SUV

## Abstract

**Background:**

Today, the standardized uptake value (SUV) is essentially the only means for quantitative evaluation of static [^18^F-]fluorodeoxyglucose (FDG) positron emission tomography (PET) investigations. However, the SUV approach has several well-known shortcomings which adversely affect the reliability of the SUV as a surrogate of the metabolic rate of glucose consumption. The standard uptake ratio (SUR), i.e., the uptake time-corrected ratio of tumor SUV to image-derived arterial blood SUV, has been shown in the first clinical studies to overcome most of these shortcomings, to decrease test-retest variability, and to increase the prognostic value in comparison to SUV. However, it is unclear, to what extent the SUR approach is vulnerable to observer variability of the additionally required blood SUV (BSUV) determination. The goal of the present work was the investigation of the interobserver variability of image-derived BSUV.

**Methods:**

FDG PET/CT scans from 83 patients (72 male, 11 female) with non-small cell lung cancer (*N* = 46) or head and neck cancer (*N* = 37) were included. BSUV was determined by 8 individuals, each applying a dedicated delineation tool for the BSUV determination in the aorta. Two of the observers applied two further tools. Altogether, five different delineation tools were used. With each used tool, delineation was performed for the whole patient group, resulting in 12 distinct observations per patient. Intersubject variability of BSUV determination was assessed using the fractional deviations for the individual patients from the patient group average and was quantified as standard deviation (SD _*is*_), 95% confidence interval, and range.

Interobserver variability of BSUV determination was assessed using the fractional deviations of the individual observers from the observer-average for the considered patient and quantified as standard deviations (SD _*p*_, SD _*d*_) or root mean square (RMS), 95% confidence interval, and range in each patient, each observer, and the pooled data respectively.

**Results:**

Interobserver variability in the pooled data amounts to RMS = 2.8% and is much smaller than the intersubject variability of BSUV (SD _*is*_= 16%). Averaged over the whole patient group, deviations of individual observers from the observer average are very small and fall in the range [ − 0.96, 1.05]%. However, interobserver variability partly differs distinctly for different patients, covering a range of [0.7, 7.4]% in the investigated patient group.

**Conclusion:**

The present investigation demonstrates that the image-based manual determination of BSUV in the aorta is sufficiently reproducible across different observers and delineation tools which is a prerequisite for accurate SUR determination. This finding is in line with the already demonstrated superior prognostic value of SUR in comparison to SUV in the first clinical studies.

## Background

Today, the standardized uptake value (SUV), defined as the tracer concentration at a certain time point normalized to injected dose per unit body weight, is essentially the only means for quantitative evaluation of static [^18^F-]fluorodeoxyglucose (FDG) positron emission tomography (PET) investigations. However, the SUV approach has several well-known shortcomings, notably, uptake time dependence of the SUV, interstudy variability of the arterial input function (AIF), and susceptibility to errors in scanner calibration [1–3], which adversely affect the reliability of the SUV as a surrogate of the metabolic rate of glucose consumption. This possibly explains the unsatisfactory performance of SUV-based therapy outcome prediction for various tumor diseases [4–16]. In recent publications, we were able to show that the uptake time-corrected ratio of tumor SUV to (image-derived) blood SUV (standard uptake ratio (SUR)) overcomes most of these shortcomings [17, 18], decreases test-retest variability [19], and increases the prognostic value compared to SUV in patients with esophageal carcinoma [20, 21] and non-small cell lung cancer [22].

While the assumptions underlying the SUR concept [17, 18] are sound, reliability of the image-based blood SUV (BSUV) determination required for SUR computation might be questioned. In our previous clinical studies [20–22], BSUV was consistently determined by the strategy described in the “Materials and methods” section and used for SUR computation. The observed superior performance of SUR in comparison to SUV demonstrates that insufficient accuracy of BSUV determination was not a critical issue in these studies. However, in all these investigations, the same individual determined BSUV with the same delineation tool and it is conceivable that reliability of BSUV is distinctly inferior when it is determined by different observers with the same or a different delineation tool. Both systematic as well as random interobserver differences would obviously limit the usefulness of SUR in longitudinal as well as cross-sectional clinical studies.

Consequently, the goal of the present work was the investigation of the interobserver variability of image-derived BSUV within single patients and across a substantial patient group. For this purpose, 8 observers from 6 institutions determined BSUV in image data from 83 patients using one or more of five different delineation tools.

## Materials and methods

### Patient group and data acquisition

The investigated patient group included 83 patients (72 male, 11 female, mean age 59.5 years, range 37–84). Data were acquired prospectively from August 2005 to August 2009 at the University Hospital, Technische Universität Dresden, in the context of two different studies (ClinicalTrials.gov identifier: NCT00180245, patients with head and neck squamous cell carcinoma (HNSCC), *N* = 37 and ClinicalTrials.gov identifier: NCT00180154, patients with non-small cell lung cancer (NSCLC), *N* = 46) and were evaluated retrospectively in the present study. All patients included in the prospective studies were also included here. Retrospective evaluation of the data was approved by the local Clinical Institutional Review Board and complies with the Declaration of Helsinki.

All patient underwent a ^18^F-FDG hybrid PET/CT scan performed with a Biograph 16, Siemens Medical Solutions Inc., Knoxville, TN, USA (3D acquisition, 3-min emission per bed position). Data acquisition started 80 ± 15.2 min after injection of 249 to 412 MBq ^18^F-FDG. All patients had fasted for at least 6 h prior to FDG injection. Tomographic images were reconstructed using attenuation-weighted OSEM reconstruction (four iterations, eight subsets, 5-mm FWHM Gaussian filter).

### BSUV determination

For the determination of the arterial blood SUV, the observers were asked to proceed as follows:

1. Select a transaxial CT image in the descending aorta immediately below the aortic arch
2. Define a circular ROI at the center of the aorta in this CT image. Adjust radius to keep approximately 8 mm away from the aortic wall. Step through consecutive planes along the descending aorta and repeat ROI definition. Skip the plane in case of
  Visible spill in into the aorta from adjacent “hot” structures
  Visible attenuation correction artifacts affecting the aorta
3. Exclude planes near and below the diaphragm (which are susceptible to motion-induced attenuation artifacts)
4. Process a sufficient number of planes to obtain a total ROI volume of at least 5 ml. If the minimum volume cannot be achieved in the descending aorta alone, delineation can be extended to the ascending aorta
5. Review the final delineation and verify its integrity regarding the mentioned exclusion criteria
6. Copy the resulting ROI to the corresponding PET data and compute BSUV as the mean value of the aorta ROI

**Fig. 1:**
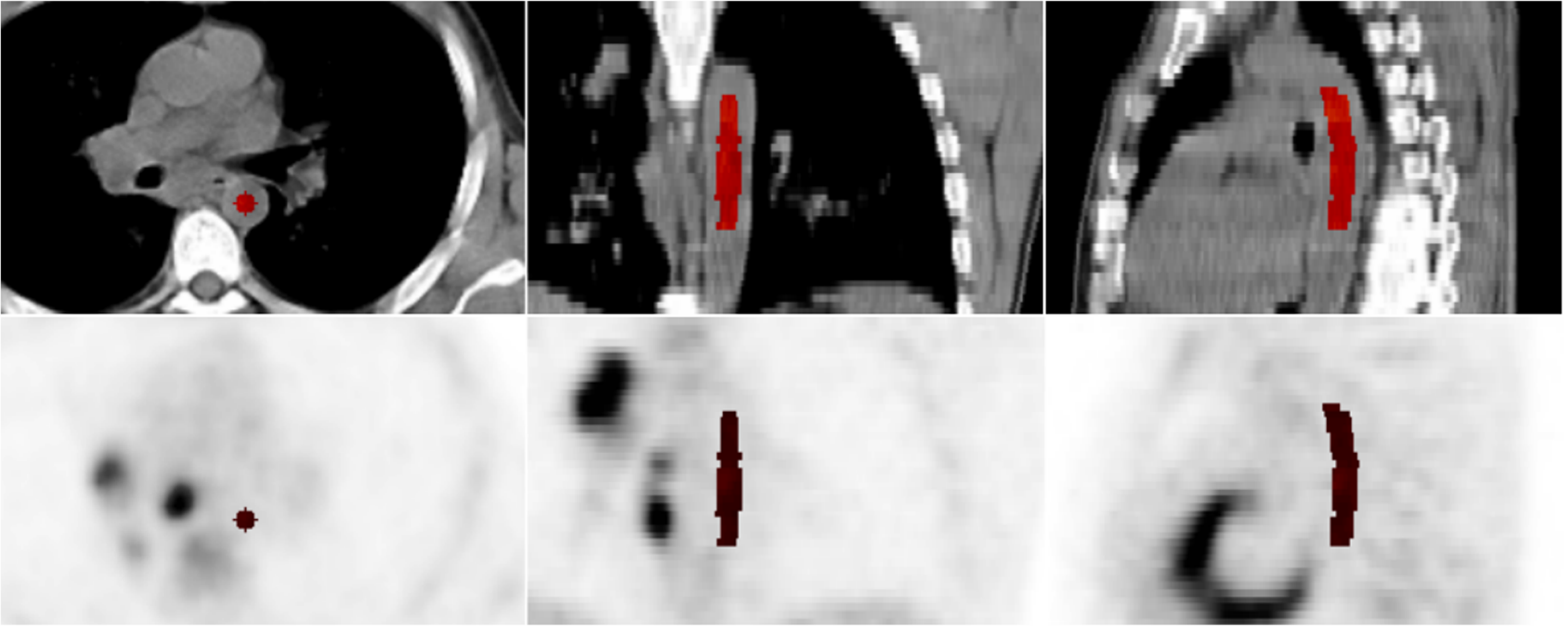
Example of a valid aorta ROI delineation (highlighted in red) observing the prescription described in the “Materials and methods” section

Figure 1 shows an example of a valid delineation.

The observers were free to use a delineation tool of their choice for the delineation task. The required time for a single data set was below 5 min with all used delineation tools. Overall, delineation was performed by eight observers using five different delineation tools. Each chosen tool was applied to the whole patient group by the observer. Six individuals used a single tool, and two individuals used three different tools, resulting in a total of *D*=12 delineations for each of *P*=83 patients, see Table 1. In the following, we denote the individually derived values as BSUV_*dp*_(*d*=[1 −− *D*],*p*=[1 −− *P*] where *p* enumerates the patients and *d* enumerates the observer/delineation tool combinations). In the following, we simply use the term “observer” to denote the different observer/delineation tool combinations.

**Table 1:** Overview of the software tools used for aorta delineation

Software	Versions	No. of observers
EBW; Philips Healthcare Best, The Netherlands	4.0.3.5	3
MIM; MIM Software Inc. Cleveland, OH, USA	6.7.6	1
OsiriX; Pixmeo SARL Bernex Switzerland	9.5; 10.0	2
PMOD; PMOD Technologies LCC Zurich, Switzerland	3.905; 3.804	3
ROVER ABX; GmbH Radeberg, Germany	3.0.32; 3.0.36	3

The third column shows the number of observers who applied the respective software to the whole patient group

### Data evaluation

The observer-averaged BSUV

$$\overline{\text{BSUV}}_{p} = \frac{1}{D} \sum_{d=1}^{D} {\text{BSUV}_{dp}} $$

was used as the best available estimator of the true (observer) population mean (the theoretical value resulting from averaging over infinitely many observers performing the delineation for this patient). Description of the intersubject variability of this quantity was based on the fractional deviation of individual patients from the patient group average $\overline {\text {BSUV}} = \frac {1}{P} \cdot \sum _{p=1}^{P} \overline {\text {BSUV}}_{p}$:

$$\Delta\overline{\text{BSUV}}_{p} = \frac{\overline{\text{BSUV}}_{p} - \overline{\text{BSUV}}}{\overline{\text{BSUV}}}\,. $$

Intersubject variability was quantified as standard deviation (SD_*is*_), 95% confidence interval (CI), and range of $\Delta \overline {\text {BSUV}}_{p}$.

Assessment of interobserver variability of BSUV determination was based on the fractional deviation of the individual observers from the respective $\overline {\text {BSUV}}_{p}$:

1 $$  \Delta\text{BSUV}_{dp} = \frac{\text{BSUV}_{dp} - \overline{\text{BSUV}}_{p}}{\overline{\text{BSUV}}_{p}}\,.  $$

Interobserver variability was quantified as standard deviation, 95% CI, and range of *Δ*BSUV_*dp*_ separately for each patient and each observer, respectively. In the pooled group of all patients and observers, the standard deviation is replaced by the root mean square (RMS) deviation for description of the width of the distribution since it follows from Eq. 1 that the mean $\Delta \overline {\text {BSUV}}$ (the average over all observers and patients) is exactly zero:

2 $$ \text{RMS} = \sqrt{\frac{1}{D \cdot P} \sum_{d=1}^{D} \sum_{p=1}^{P} \Delta\text{BSUV}_{dp}^{2}}\,.   $$

The relevant standard deviations are given by

3 $$ \text{SD}_{p} = \sqrt{\frac{1}{D - 1} \sum_{d=1}^{D} \left (\Delta\text{BSUV}_{dp} -{\overline{\Delta\text{BSUV}}_{p}} \right)^{2}}   $$

where

$$\overline{\Delta\text{BSUV}}_{p} = \frac{1}{D} \sum_{d=1}^{D} \Delta\text{BSUV}_{dp} $$

is the observer-averaged *Δ*BSUV for patient *p* and

4 $$ \text{SD}_{d} = \sqrt{\frac{1}{P - 1} \sum_{p=1}^{P} \left (\Delta\text{BSUV}_{dp} -\overline{\Delta\text{BSUV}}_{d} \right)^{2}}   $$

where

$$\overline{\Delta\text{BSUV}}_{d} = \frac{1}{P} \sum_{p=1}^{P} \Delta\text{BSUV}_{dp}\,, $$

is the patient-averaged *Δ*BSUV for observer *d*.

SD _*p*_ thus measures interobserver variability separately in each patient while SD _*d*_ allows to compare the performance of different observers.

Data analysis was performed with the *R language and environment for statistical computing* [23] version 3.5.0.

## Results

**Fig. 2:**
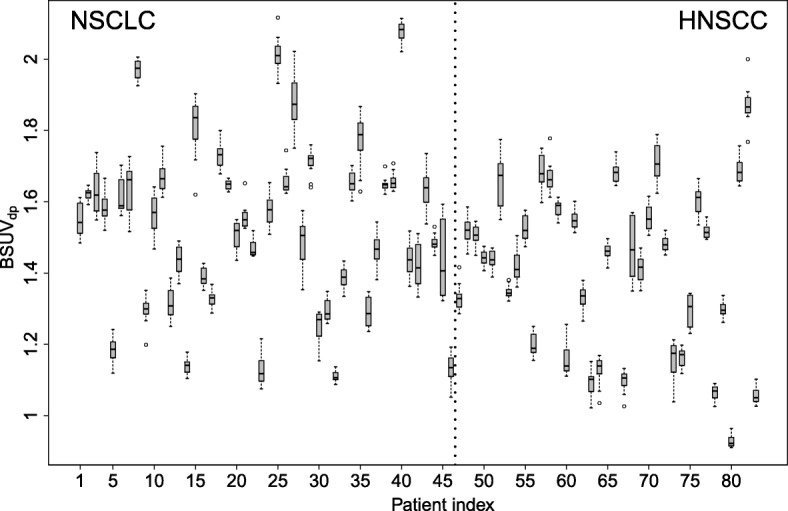
Boxplot of the observed blood SUV (BSUV_*dp*_), grouped by patient. Note that intersubject variability is much larger than interobserver variability for each patient

**Fig. 3:**
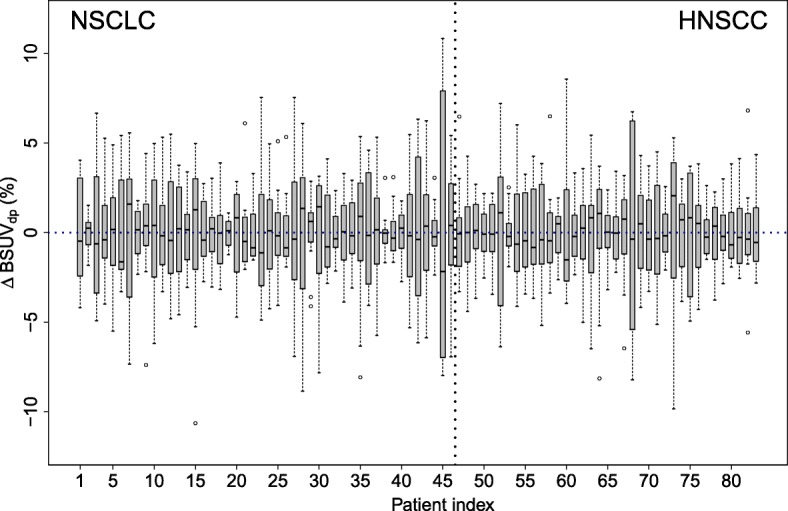
Boxplot of fractional deviation from observer mean for the respective patient (*Δ*BSUV_*dp*_), grouped by patient. Note the patient dependence of the magnitude of the interobserver variability

**Fig. 4:**
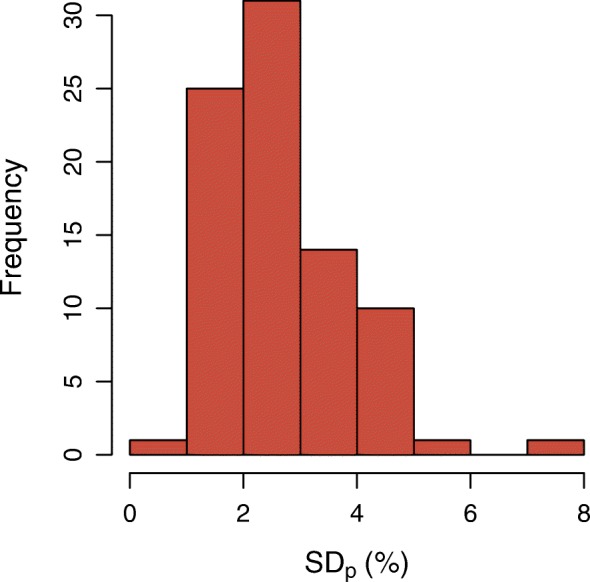
Histogram of patient-specific interobserver variability, described by SD _*p*_ (Eq. 3), the standard deviation of the distribution of fractional deviations *Δ*BSUV_*dp*_ (Eq. 1) from observer mean for the respective patient grouped by patient as illustrated in Fig. 3

**Fig. 5:**
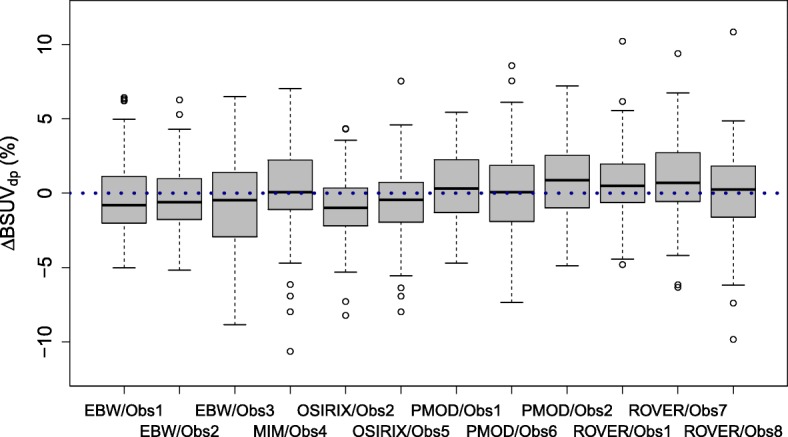
Boxplot of fractional deviation from observer mean for the respective patient (*Δ*BSUV_*dp*_), grouped by observer. Note the comparable performance of all observers

**Fig. 6:**
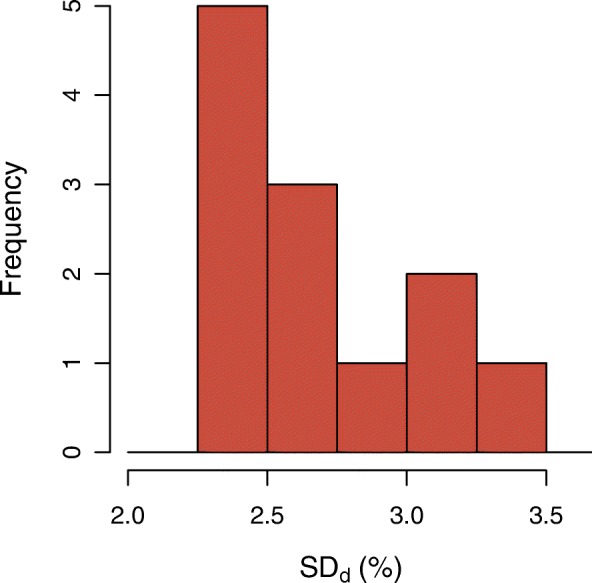
Histogram of observer performance contribution to the interobserver variability, described by SD _*d*_ (Eq. 4), the standard deviation of the distribution of fractional deviations *Δ*BSUV_*dp*_ (Eq. 1) from observer mean for the respective patient grouped by observer as illustrated in Fig. 5

**Fig. 7:**
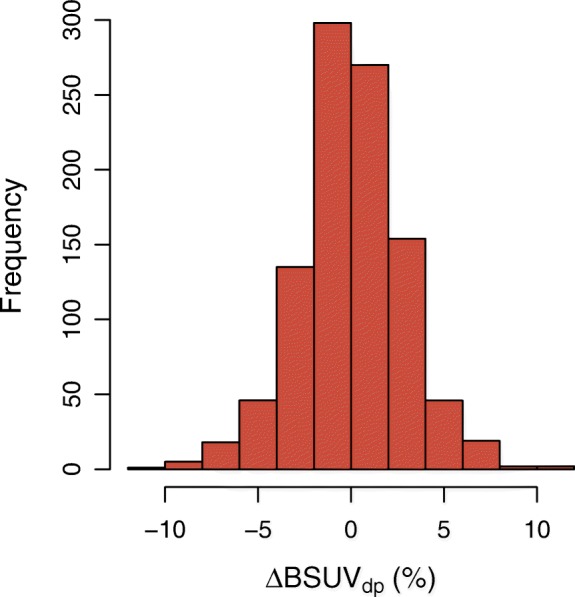
Histogram of pooled interobserver variability, *Δ*BSUV_*dp*_, expressed as fractional deviation from observer mean for the respective patient (see Eq. 1)

A boxplot of the observed BSUV_*dp*_ grouped by patient is shown in Fig. 2. The corresponding boxplot of *Δ*BSUV_*dp*_ is shown in Fig. 3. There is a clear patient dependence of the interobserver variability as signaled by the variable interquartile ranges in these plots. A pairwise comparison of the variances of the corresponding distributions revealed in 30% of the comparisons a significant difference (*P* < 0.05) according to a two-tailed *F* test. This patient dependence is further illustrated in Fig. 4 which shows the frequency distribution of SD _*p*_. A boxplot of the derived *Δ*BSUV_*dp*_ grouped by observer is shown in Fig. 5. Averaged over the whole patient group, the individual observers differ only slightly (range [ − 0.96, 1.05]%) from the observer average (although the difference reaches statistical significance in 5 out of 12 observers according to a two sided Mann-Whitney test). No significant difference of the variances of the corresponding distributions was found in a pairwise comparison. Figure 6 shows the corresponding SD _*d*_ distribution which demonstrates the (small) differences in observer performance. Finally, Fig. 7 shows the histogram of the complete pooled *Δ*BSUV_*dp*_ data. The relevant quantitative measures are summarized in Table 2.

**Table 2:** Intersubject and interobserver variability of BSUV described by the quantities defined in Eqs. 1–4 (at the stated accuracy level, RMS of *Δ*BSUV_*dp*_ according to Eq. 2 is identical to the standard deviation)

	Mean	Standard deviation	95% CI	Range
$\Delta \overline {\text {BSUV}}_{p}$	0	16.0	[ − 27.7, 33.7]	[ − 37.0, 41.0]
*Δ*BSUV_*dp*_	0	2.77	[ − 5.87, 5.50]	[ − 10.6, 10.8]
SD _*d*_	2.69	0.38	[2.29, 3.40]	[2.29, 3.40]
SD _*p*_	2.66	1.14	[1.14, 4.95]	[0.74, 7.39]

All figures are specified as percentages

## Discussion

In this study, we investigated the interobserver variability of image-based BSUV determination in the aorta. In the pooled group of all observers and patients, we found an interobserver variability of *RMS*=2.8%. This figure has to be compared with an intersubject variability of (observer-averaged) BSUV of SD_*is*_=16% in the investigated patient group (which is in complete agreement with other reports [24, 25]).

Thus, our main result is that interobserver variability of manually determined BSUV is much smaller (by nearly a factor of six) than the typical intersubject variability of this quantity and has, therefore, no relevant negative effect on assessment of true intersubject variability of BSUV. Regarding the use of image-derived BSUV in SUR computation, this finding demonstrates that validity of the SUR approach is not compromised by observer-induced uncertainties of BSUV determination. It should be emphasized that it is of no concern in this context, whether part of the observed substantial intersubject variability of BSUV is possibly caused by imperfections of SUV calibration of the considered PET system and/or trivial errors such as erroneous dose or body weight since any such effect causes a global rescaling of the image data and will thus cancel in computation of SUR.

As demonstrated by our data, it is, however, relevant to ensure that the evaluated portions of the reconstructed images are free of spurious changes of the lesion to blood image contrast which might be caused by attenuation and scatter correction related effects in certain regions, notably induced by organ motion near the diaphragm and liver dome. Indeed, while the overall interobserver variability in the investigated patient group is very small, closer inspection of the data on a per-patient basis revealed that some patients exhibit substantially increased interobserver variability (see Figs. 2 and 3). Consequently, the SD _*p*_ histogram in Fig. 4 shows a tail towards higher SD _*p*_ values in a small fraction of patients. Retrospective examination of the affected image data identified in most of them spurious, motion-induced signal decrease due to attenuation undercorrection and/or scatter overcorrection (caused by attenuation/emission mismatch near the liver dome). This signal drop also affects part of the aorta, and the affected areas were erroneously not excluded from delineation by some observers (thus deviating from the provided procedure guideline). Such sporadic oversights are possibly unavoidable, as their occurrence in the present study suggests. It might therefore be advisable to exclude the potentially affected region categorically (instead of letting the observer decide this on a per case basis) by not extending delineation below a plane about 5 cm above the diaphragm. But even with the presently used prescription, the worst case deviation from the observer mean for any patient remained below 11% which still is much smaller than the observed BSUV intersubject variability (range [ − 37, 41]%). Nevertheless, a clear patient dependence of the interobserver variability as described by SD _*p*_ is present which has a range equal to [0.7, 7.4]%. In comparison, the overall performance of the different observers when averaged over the whole patient group is rather similar as illustrated by Fig. 5 and the small SD _*d*_ range of [2.3, 3.4]%.

A potential shortcoming of the present study is the limited number of observers and delineation tools included. However, considering the very consistent performance of all observers and software tools regarding variability and deviation from the observer average, the obtained results are statistically already sufficiently reliable in our view. Therefore, our results overall demonstrate a very low interobserver variability of image-derived BSUV. Theoretically, the obtained BSUVs could still be negatively biased by partial volume effects (which would lead to systematic errors when computing SURs). However, by using a prescribed safety margin of about 8 mm to the aortic wall, partial volume effects are reduced to a negligible level. Even for a rather pessimistic scenario with a combination of small luminal aorta diameter of 21 mm [26, 27] and low spatial resolution in the image data of FWHM=8 mm, signal recovery of delineation-averaged BSUV in a straight cylinder is equal to 0.985.

## Conclusion

The present investigation demonstrates that the image-based manual determination of BSUV in the aorta is sufficiently reproducible across different observers and delineation tools which is a prerequisite for accurate SUR determination. This finding is in line with the already demonstrated superior prognostic value of SUR in comparison to SUV in the first clinical studies. The next logical step will be to fully automatize BSUV determination for a more streamlined use of SUR in the clinical setting. The presented data might serve as a valuable resource for validation of such future algorithms.
